# A review on guided bone regeneration using titanium mesh

**DOI:** 10.6026/973206300200562

**Published:** 2024-05-31

**Authors:** Saad Obaid Alazmi

**Affiliations:** 1 Department of Periodontology and Implant Dentistry, College of Dentistry, Qassim University, Saudi Arabia

**Keywords:** Alveolar bone, Guided bone regeneration, Implant, Titanium mesh

## Abstract

The gold standard for bone regeneration in atrophic ridge patients is guided bone regeneration (GBR). This makes it possible to get enough bone volume for an appropriate implant-prosthetic rehabilitation. The barrier membranes must meet the primary GBR design requirements, which include adequate integration with the surrounding tissue, spaciousness and clinical manageability. Titanium mesh's superior mechanical qualities and biocompatibility have broadened the indications of GBR technology, enabling it to be used to restore alveolar ridges with more significant bone defects. GBR with titanium mesh is being used in many clinical settings and for a range of clinical procedures. Furthermore, several advancements in digitalization and material modification have resulted from the study of GBR using titanium mesh. Hence, we report a review on the various characteristics of titanium mesh and its current use in clinical settings for bone augmentation.

## Background:

Atropic ridge resorption frequently results in insufficient bone for implant implantation [1- 2]. In conjunction with implant treatment, the GBR surgery seems to be one of the most popular and dependable methods for restoring bone abnormalities in height and/or width [3- 4]. GBR stimulates multidirectional osteogenesis and supports osteogenic stability. The GBR approach combines bone substitute materials with either resorbable or non-resorbable membranes, depending on the type of bone defect [5]. While bone-graft materials guide and control the proliferation and development of osteoblastic progenitor cells, barrier membranes prevent early epithelial and connective cells from colonizing the region [6-7]. Elasticity may lessen the compression of the oral mucosa, stiffness and strength aid in the osteogenesis process and stability allows bone-filling materials to retain their volume during healing [8-9]. GBR bone augmentation can be performed separately or in conjunction with implant placement [10-11]. Through histological and histomorphometric research, Andreasi *et al.* proved the efficacy of directed bone regeneration using titanium mesh as a barrier membrane [12]. Because standardized meshes contain preset properties like thickness and width, they need to be manually modeled in order to fit the patient's unique alveolar ridge. Numerous problems with this approach include extended surgical times, inaccurate fitting, pain, infection, laceration of the flap, and potential mesh exposure in the future [13]. With the aid of 3D digital models and pre-operative cross-sectional imaging [cone beam computed tomography (CBCT)], the patient's alveolar ridge can be digitally recreated using computer-aided reconstruction (CAD) technology [14].

## Customized titanium mesh:

Because standardized meshes are products with predetermined properties like thickness and width, they need to be manually modelled in order to fit the patient's alveolar ridge precisely. Numerous problems with this approach include extended surgical times, inaccurate fitting, discomfort, infection, laceration of the flap, and potential mesh exposure in the future [15, 16]. With the collection of pre-operative cross-sectional imaging (CBCT) and 3D digital models, the patient's alveolar ridge can be digitally recreated using CAD technology [17].

Important mechanical characteristics of titanium mesh include good bending and shape flexibility as well as a strong, stable osteogenesis effect [18]. Porosity and thickness are important considerations when using titanium mesh; a 0.2 mm mesh can be flexible enough to work in most circumstances. Numerous research endeavours have tried to explore the function of titanium mesh porosity, with sometimes contentious outcomes. It is not quite evident how pore size and the pace of soft tissue growth relate to one another. Titanium resists corrosion very well and continuously [19]. Following bone repair, a thin layer of tissue, known as the "pseudo-periosteum" is discovered to be present on the regenerating bone surface. This pseudo-periosteal may help to avoid infections and safeguard grafts. Strong osteogenesis prediction is a feature of GBR with titanium mesh, and both horizontal and vertical bone augmentation can be achieved with simultaneous or delayed implantation. The typical bone augmentation for the delayed implantation approach was 4-5 mm in bone width and 5-7 mm in bone height [19].

Since titanium mesh cannot be reabsorbed by the body, patients must undergo traumatizing second-stage surgery to remove both the titanium mesh and the fixation screws. On the other hand, the intraoperative risk of contamination, handling, and trimming is eliminated by designing and printing a customized mesh that mimics the optimum reconstruction, which shortens the surgical time and lessens the strain on the soft tissues. Since 3D laser-sintering printing technology allows for regular and rounded edges as well, this is also applicable [20]. Various procedures can be used to manufacture custom-made titanium mesh, such as mesh-preforming on patients' 3D jaw models or CAD/CAM3D printing [21]. Thus, customized meshes allow for more rapid surgery, more precise fitting, fewer pins needed to secure the mesh, smoother edges, and ultimately reduced mucosal stress [22].

## 3D Technology, bone defect dimension and GBR:

A customized grid for GBR, for bone deformities, with the right physical and biological qualities has been made possible by the application of 3D technology and additive manufacturing processes. The most frequent complication following GBR surgery, mucosal rupture and subsequent mesh exposure, are decreased by the smooth edges of the 3D-printed Ti-mesh, in contrast to regular Ti-mesh. Additionally, it can be built more precisely to fit the surgical site's bone abnormalities [22, 23]. Whether the bone defects are major or little, the 3D precision of the bone increase is not considerably correlated with the size of the bone shortfall. With virtual planning and patient-specific CAD/CAM mesh production, large combined alveolar bone deficiencies in both horizontal and vertical dimensions could be safely and predictably repaired at the same time as implants [24]. The bespoke mesh is shaped using 3D patient models, which encourage proper alveolar bone adaption. Up to 90% bone regeneration rate is an effective treatment for vertical bone insufficiency. Research has shown that patient-specific Ti-mesh can potentially support significant bone augmentation in complex bone defects up to 11.48 mm in horizontal and 8.90 mm in vertical dimensions. This suggests that laser-sintered CAD/CAM mesh is a dependable substitute for traditional bone grafting procedures when treating extended atrophic alveolar ridges [25].

## Customized titanium mesh and aesthetics:

Reconstruction becomes necessary because maxillary and mandibular abnormalities caused by trauma, tumours, or congenital diseases have a substantial impact on an individual's functional and aesthetic quality. The customized grid makes it possible to obtain and reinforce 3D bone repair, restoring the defect's functional and aesthetic aspects and maintaining the contour of the bone in the process. They improve the accuracy of bone augmentation and maxillary connectivity by making it easier to correctly position graft material to install implant fehixtures [26]. An appropriate volume of alveolar bone is needed for the implant in the front maxillary aesthetic zone. Individualized mesh therefore contributes to encouraging outcomes. Virtual bone volume augmentation and the creation of tailored titanium mesh using 3D printing technology resulted in a significant increase in bone (3.7 mm SD 0.59 at 6 months and 4.3 mm (SD 0.83) at 12 months) in patients with vestibular bone concavities. Focus should be placed on achieving optimal soft tissue management, such as voluminous and healthy tissue, as well as good aesthetic without any indications of fibrosis or scarring, while putting a customized 3D Ti-mesh. Moreover, prosthetic ally guided regeneration (PGR) in conjunction with customized titanium mesh assists in overlaying a digital diagnostic wax-up to facilitate guided bone restoration and maintain a sufficient buccal cortical to guarantee a satisfying cosmetic result. [26, 27]

## Histological picture:

According to studies, histological examination of the regenerated sites revealed the existence of freshly formed, mineralized bone developing beneath the titanium mesh. Additionally, several specimens displayed a layer of connective tissue in the biopsy's most coronal region [28, 29, 30 and 31]. According to Andreasi *et al.*, the samples' histological and histomorphometric analyses showed how well directed bone regeneration using titanium mesh as a barrier membrane worked [12]. According to Cucchi *et al.*, the formed bone's trabecular organization was distinct from that of the native bone, and the newly created bone remained juvenile and dissimilar to the native bone [32]. Utilising customized and digitized meshes, it was possible to observe the mineralization of the regenerated, enhanced alveolar bone next to the remnants of bone substitute materials in the medullary cavities or connective tissue. A newly regenerated tissue with structure, organization, vitality, and functional processes of remodelling and assimilation of grafting materials was observed without any indications of inflammation. Additionally, re-epithelialization beneath the mesh's intern section was shown to occur quickly and naturally [32].

## Customized mesh and clinical success:

To attain clinical success in the GBR technique, virtual planning and customized grid manufacturing related to flap arrangement and its control are essential factors to take into account. Because customized Ti-meshes are stiffer than ordinary ones, mesh exposure may still occur after this digital process. This could be the result of the flap of mucosal tissue being mechanically stressed, the placement of a removable prosthesis following surgery, or finally the learning curve of the digital software and the grid-projecting processes. Resorbable membrane application over customized mesh may lower healing problem rates (13.3% vs. 33.3%) [33].

## Early and late clinical complication of customized mesh and its management:

Even with mesh exposure, GBR success is still possible with the right management. Pharmacological or mechanical methods are used in treatment. When mesh exposure develops four weeks following surgery, it is typically treated with gels containing 0.2% chlorhexidine (CHX), administered twice or four times a day, and then the relevant region is curetted until the tissue heals. As an alternative, the literature also recommends CHX mouthwashes or CHX spray with variable strengths for CHX gel applications. But gel formulations appear to work better than mouthwashes [34]. Topical antibiotic treatment becomes important for treating graft infection suspected, however literature rarely reports antibiotic medication when mesh exposure occurs. Due to pus and infection, this situation demands the rapid removal of the mesh. During these phases, maintaining good oral hygiene and controlling plaque are also essential [35]. Applying CHX 0.2% or, in certain circumstances, 1% gel twice a day until tissue recovery seems beneficial and allows the mesh to be maintained is a good strategy for managing late exposure. Secondary wound healing can be facilitated by mechanically polishing the mesh edges with carbide or diamond burs for late exposure [36, 37]. Louis *et al.* discovered that 23 meshes had been exposed on 44 patients (52%) with a distinct graft failure and 97% of the instances showing successful bone grafting [38]. On the other hand, exposure of the mesh caused early resorption of the site in 15% and 25% of cases in a research by Maiorana *et al.* (2015) [39].

## Conclusion:

The use of a titanium mesh in conjunction with GBR is a reliable and safe method for treating bone deficiencies surrounding dental implants by increasing alveolar bone volume. However, longer recovery times and higher patient morbidity due to the need for a second-stage surgical procedure are drawbacks. Moreover, there is a significant chance of membrane exposure and soft tissue dehiscence. Nonetheless, it is a viable technique for reconstructing the alveolar ridge because of the remarkable regeneration and implant stability outcomes.

## References

[1] Hansson S (2012). J Dent Biomech..

[2] Tan WL (2012). Clin Oral Implants Res..

[3] Elgali I (2017). Eur J Oral Sci..

[4] Bornstein MM (2008). Int J Oral Maxillofac Implants..

[5] Retzepi M (2010). Clin Oral Implants Res..

[6] Ortega-Oller et (2015). Biomed Res Int..

[7] Kormas I (2022). Membranes (Basel)..

[8] Jung G (2014). J Korean Assoc Oral Maxillofac Surg..

[9] Badhey A (2017). Semin Plast Surg..

[10] Zhou M (2018). J Oral Implantol..

[11] Barreta M (2020). Materials (Basel)..

[12] Andreasi Bassi M (2016). Oral Implantol (Rome)..

[13] Hartmann A (2019). Implant Dent..

[14] Al-Ardah AJ (2018). Oral Implantol..

[15] Okubo T (2018). J Oral Sci..

[16] Andrea Scribante A (2023). Prosthesis..

[17] Yen HH (2018). Curr Oral Health Rep..

[18] Hasegawa H (2019). Int J Oral Maxillofac Implants..

[19] Sidambe A.T. (2014). Materials..

[20] Chiapasco M (2021). Clin. Oral Implant. Res..

[21] Hartmann A (2020). BMC Oral Health..

[22] Ciocca L (2011). Med. Biol. Eng. Comput..

[23] Windisch P (2021). Clin. Oral Investig..

[24] Yang W (2022). BMC Oral Health..

[25] De Santis D (2022). J Clin Med..

[26] Iino M (2009). Oral Pathol. Oral Radiol. Endod..

[27] Farronato D (2020). Materials..

[28] Chiapasco M (2018). Periodontol 2000..

[29] Maiorana C (2021). J Contemp Dent Pract..

[30] Torres J (2010). J Clin Periodontol..

[31] Corinaldesi G (2007). The Journal of Periodontology..

[32] Cucchi A (2021). Clin Oral Implants Res..

[33] De Santis D (2021). Medicina (Kaunas)..

[34] Canullo L (2020). Int. J. Oral Maxillofac. Implant..

[35] Roccuzzo M (2004). Clin. Oral Implant. Res..

[36] Al-Ardah A.J (2017). J. Oral Implantol..

[37] Her S (2012). J. Oral Maxillofac. Surg..

[38] Louis PJ (2008). J Oral Maxillofac Surg..

[39] Maiorana C (2021). J Contemp Dent Pract..

